# Diversity and Biocontrol Potential of Culturable Endophytic Fungi in Cotton

**DOI:** 10.3389/fmicb.2021.698930

**Published:** 2021-08-13

**Authors:** Lirong Jin, Long Yang, Wenjing Li, Dong Xu, Nina Yang, Guoqing Li, Peng Wan

**Affiliations:** ^1^State Key Laboratory of Agricultural Microbiology, Key Laboratory of Plant Pathology of Hubei Province, Huazhong Agricultural University, Wuhan, China; ^2^Key Laboratory of Integrated Pest Management Crops in Central China, Ministry of Agriculture, Hubei Academy of Agricultural Sciences, Wuhan, China; ^3^Hubei Key Laboratory of Crop Disease, Insect Pests and Weeds Control, Plant Protection, Soil and Fertilizer Research Institute, Hubei Academy of Agricultural Sciences, Wuhan, China

**Keywords:** cotton, *Verticillium dahliae*, endophytic fungi, *Fusarium proliferatum* 10R-7, biological control

## Abstract

Healthy cotton samples were collected and 93 endophytic fungal strains were isolated: 23 strains from the roots and 70 strains from the stems. Morphological characterization and ITS sequence analysis were used for the identification of these isolates. The results showed that the 93 strains including 20 species were highly diverse in terms of their taxonomy. Simpson’s and Shannon’s diversity indices were 0.915 and 3.848, respectively. *Fusarium* and *Alternaria* were the two dominant genera, constituting 19.4% of the total strains. Then, 72 spore-producing strains were tested for the suppression of cotton *Verticillium* wilt (CVW) caused by *Verticillium dahliae* in a greenhouse. Five strains exhibited effective suppression of CVW with average efficacy values higher than 50%. One of the effective strains, namely, *Fusarium proliferatum* 10R-7, was selected for the investigation of the role of fusaric acid, a secondary metabolite of strain 10R-7, in the suppression of *V. dahliae* and CVW. The results showed that *F. proliferatum* 10R-7 could produce fusaric acid, and this metabolite exhibited 100% inhibition of mycelial growth of *V. dahliae* at concentrations higher than 20 μg/ml. However, fusaric acid at 2.5 to 80 μg/ml was not effective in the suppression of CVW, compared with the control treatment with *V. dahliae* alone. *F. proliferatum* 10R-7 was labeled with green fluorescent protein (GFP), and the GFP-tagged strain was found to be able to colonize inside the taproots of cotton, suggesting that *F. proliferatum* 10R-7 is a true endophyte of cotton and endophytic colonization may play a role in the suppression of infection of cotton by *V. dahliae*.

## Introduction

Cotton is an economically important crop grown worldwide. Cotton *Verticillium* wilt caused by *Verticillium dahliae* Kleb. is a plant disease that leads to severe economic losses in cotton (Ma et al., 2002). As a soilborne plant disease, it is difficult to control it utilizing conventional fungicide (Berg et al., 2001). Meanwhile, *V. dahliae* could produce dormant structures called microsclerotia with high stress resistance. The microsclerotia can persist in soil for over 14 years (Klosterman et al., 2009), which increase the difficulty of controlling the disease. The method of controlling the disease includes breeding of resistant cultivars, change of agricultural planting pattern, and chemical control (Aguado et al., 2008; Erdogan and Benlioglu, 2010). However, these methods are not so effective or not environmentally friendly as expected (Erdogan and Benlioglu, 2010).

Biological control is one of the most promising approaches for suppressing *Verticillium* wilt because of its being environmentally friendly. Some fungi and bacteria have been reported as biocontrol agents (BCAs) against *V. dahliae* (Berg et al., 2001, 2005; Mercado-Blanco et al., 2004; Malandraki et al., 2008; Uppal et al., 2008; Erdogan and Benlioglu, 2010; Zheng et al., 2011; Deketelaere et al., 2017). There were also numerous reports about the biocontrol potential of *Fusarium* spp. to suppress *V. dahliae* (Deketelaere et al., 2017). These *Fusarium* spp. include *F. culmorum* (Dutta, 1981), *F. lateritium* (García et al., 2011), *F. moniliforme* (Varo et al., 2016a,b), *F. solani* (Zheng et al., 2011), and *F. oxysporum* (Díaz et al., 2005; Malandraki et al., 2008; Pantelides et al., 2009; Gizi et al., 2011; Veloso and Díaz, 2012; Angelopoulou et al., 2014; Zhang et al., 2015). Their primary mechanisms of action against *V. dahliae* are antibiosis (Dutta, 1981; García et al., 2011), competition for nutrients and/or ecological sites (Larkin and Fravel, 1999; Pantelides et al., 2009; Varo et al., 2016a,b), triggering of induced systemic resistance (Díaz et al., 2005; Olivain et al., 2006; Malandraki et al., 2008; Angelopoulou et al., 2014), and mycoparasitism (Nagtzaam et al., 1998). Competition included competition for nutrients and ecological sites on the root (Gizi et al., 2011; Angelopoulou et al., 2014).

Endophytic fungi are distributed widely and have been found in almost all of the plants. They are highly diversified in species composition across many plants (Rodriguez et al., 2009; Potshangbam et al., 2017). Previous studies have confirmed the diversity of cotton endophytic fungi (Ek-Ramos et al., 2013; Zhi-Fang Li et al., 2014). As a potential biocontrol resource, many endophytic fungi have the function of suppressing plant disease and insect pests, promoting growth of their host plants, and enhancing plant resistance (Backman and Sikora, 2008; Hassani et al., 2018). In the present study, we isolated 93 endophytic fungi from cotton, analyzed the diversity of the endophytic fungi, and evaluated the ability of these endophytic fungi to control cotton *Verticillium* wilt (CVW). The specific objectives were (*i*) to isolate cotton endophytic fungi and analyze the diversity of the endophytic fungi, (*ii*) to screen endophytic fungi with high biocontrol efficacy against *V. dahliae*, and (*iii*) to investigate the possible biocontrol mechanisms of *Fusarium proliferatum* 10R-7 against *V. dahliae* by identifying the antifungal metabolites of *F. proliferatum* 10R-7 and assessing its colonization in cotton roots.

## Materials and Methods

### Isolation of Endophytic Fungi

One hundred and eighty healthy cotton samples were collected in the fields of Wuhan City (30°28′N, 114°21′E), Hubei Province, China. First, the plant samples were rinsed with tap water and dried with filter paper. The samples were cut into small pieces (3-cm-long segments) and surface-sterilized by submerging in 70% ethanol (v/v) for 1 min, 5% sodium hypochlorite (v/v) for 5 min, and 70% ethanol again for 30 s, followed by rinsing in autoclaved distilled water five times (Hallmann et al., 2006; Zhang et al., 2014). Then, the cotton tissues were blotted with sterilized filter paper and cut into short pieces with sterile scissors, placed in an autoclaved mortar containing 1 ml of sterile water, and triturated with an autoclaved pestle. The mixtures were plated on aPDA (PDA amended with lactic acid, pH 2.5–3.5) media and were cultured at 25°C for approximately 2 weeks until the fungal endophyte hyphae grew. The fungal colonies were individually transferred to new PDA dishes, with one colony per dish, and incubated at 25°C. Finally, the fungal cultures were purified and stored at −80°C.

### Identification of the Endophytic Fungi

The endophytic fungi were identified by adopting the combined method of morphological and molecular identification. The morphological characteristics of the colonies and spores of the fungal isolates were examined (Kirk et al., 2008). Moreover, the internal transcribed spacer (ITS) region of rDNA (ITS1–5.8S rDNA–ITS2) of each isolate was amplified using the universal primers ITS1 (5′-TCC GTA GGT GAA CCT GCG G-3′) and ITS4 (5′-TCC TCC GCT TAT TGA TAT GC-3′), and the amplified products were purified, cloned, and sequenced at Sangon Bioengineering Co., Ltd., Shanghai, China (Zhang et al., 2014). The BLAST program was used to compare the resulting sequence with that of the closest related fungal species in the NCBI database. Then, the strains were identified to species by consideration of the ITS sequence and the morphological characteristics.

Several indicators for diversity assessment, including species richness (*S*), species proportion (*f*), Simpson’s diversity index (*D*), and Shannon’s diversity index (*H’*), were used to evaluate the community diversity of the endophytic isolates from cotton. The formulas are as follows:

(1) $$P\,i=\frac{n\,i}{N}$$

(2) $$D=1-\Sigma \,{\left(P\,i\right)}^{2}$$

(3) H=′-ΣPi×log2Pi

where *n*_*i*_ represents the number of isolates of the same species and *N* represents the total number of endophytic fungi.

### Screening of Biocontrol Agents in a Greenhouse

In the first round of screening, the fungi that produced spores were screened out by microscopic observation. The spore-producing isolates were further screened based on the greenhouse experiments. The method was as follows: the seeds of upland cotton (*Gossypium hirsutum*) variety “JI11,” a susceptible cultivar, were soaked in sterile water for 12 h and planted into nutrient vermiculite culture mixture (nursery substrate:sand = 1:1, v/v). When the seedlings grew to the two-true-leaf stage, the roots were uprooted and rinsed clean under tap water. The conidia of each endophytic fungus (EF) were collected from 7-day-old potato dextrose broth shake cultures and filtered through four layers of cheesecloth, followed by centrifugation to remove the supernatant. Then, the conidial precipitate was dissolved in sterile water and diluted to 1 × 10^7^ conidia/ml. The conidia of *V. dahliae* V991b were harvested by washing 15-day-old PDA cultures (at 25°C) with autoclaved distilled water, and the mixtures were filtered to remove the hyphae. Then, the conidial concentration was adjusted to 1 × 10^7^ conidia/ml for *V. dahliae* (*Vd*) and the endophytic fungus (EF). There were four treatments in this experiment: EF + *Vd*, *Vd* alone, EF alone, and sterile water control (CK). In the treatment of EF + *Vd*, the conidial suspensions of *V. dahliae* and the endophytic fungus were mixed in equal volumes to a final concentration of 5 × 10^6^ conidia/ml. In the treatments of EF alone and *Vd* alone, the conidial suspensions of EF and *Vd* were separately diluted by half with sterile water. The roots of cotton seedlings (10–15 seedlings per treatment) were dipped for 20 min in the conidial suspensions of the four treatments, and each treatment included 10–15 seedlings. Then, the seedlings were transplanted into nutrient vermiculite culture mixtures in plastic pots (12 × 10 cm, diameter × height) with three to five seedlings per pot for each treatment. The pots with the seedlings were maintained in a greenhouse (25 ± 3°C), and the seedlings were watered to maintain an appropriate humidity. At 15, 20, 25, and 30 days postinoculation (dpi), the seedlings were individually rated for severity of *Verticillium* wilt. Disease severity for each seedling was rated using a numeric scale of 0 to 4, with 0 = healthy; 1 = one to two cotyledons showing wilting symptoms, 2 = one true leaf showing wilting symptoms, 3 = two or more true leaves showing wilting symptoms, and 4 = plant dead (Shi et al., 1993). The disease index (*DI*) for each treatment was calculated on the basis of the disease severity rating and the number of cotton seedlings according to the following formula:

(4) $$D\,I=\frac{0\times n\,0+1\times n\,1+2\times n\,2+3\times n\,3+4\times n\,4}{N\times 4}\times 100$$

where *n*0, *n*1, *n*2, *n*3, and *n*4 represent the numbers of cotton seedlings with disease severity scores of 0, 1, 2, 3, and 4, respectively, and the number *N* represents the total number of tested cotton seedlings for each treatment. The biocontrol efficacy (*BE*) was calculated according to the following formula:

(5) B⁢E=[(D⁢I⁢i⁢n⁢c⁢o⁢n⁢t⁢r⁢o⁢l-D⁢I⁢i⁢n⁢E⁢F)/D⁢I⁢i⁢n⁢c⁢o⁢n⁢t⁢r⁢o⁢l]×100%

This primary screening experiment was carried out three times, and the fungal isolates screened in the primary screening were subjected to the secondary screening using the same method mentioned above.

### Control of *Verticillium dahliae* by Different Concentrations of *Fusarium proliferatum* 10R-7

The biocontrol efficacy of four conidial concentrations of *F. proliferatum* 10R-7 against *V. dahliae* was evaluated in greenhouse experiments. Seedlings of the cotton variety “JI11” were prepared as described above. Conidia of *F. proliferatum* 10R-7 were harvested by washing 7-day-old PDA cultures (25°C) with autoclaved distilled water, and the resulting mixtures were filtered and the concentration of the conidial suspension was adjusted to 1 × 10^7^, 1 × 10^6^, 1 × 10^5^, and 1 × 10^4^ conidia/ml. *V. dahliae* V991b was harvested following the method described above, and the concentration was adjusted to 5 × 10^6^ conidia/ml. The roots of cotton seedlings were submerged in 50 ml of conidial suspension of *Vd* for 20 min after being submerged in 50 ml of different concentrations of *F. proliferatum* 10R-7 for 10 min. Plants were submerged in 50 ml of the conidial suspension of *Vd* for 20 min after being submerged with 50 ml of sterile water for 10 min were used as controls. Each treatment included 10 plants, and the experiment was replicated three times. The plants were kept at 25 ± 3°C with a 14-h photoperiod in the greenhouse. The disease severity index was calculated and analyzed according to the method above.

### Bioassay of the Volatile Organic Compounds of the *Fusarium proliferatum* 10R-7

The *F. proliferatum* 10R-7 screened in the abovementioned greenhouse experiment was used in this bioassay. The double-dish method was adopted for the bioassay of the volatile organic compounds of the strain 10R-7. The *F. proliferatum* 10R-7 and *V. dahliae* V991b were incubated at opposite position to form the double dishes. The colony diameter of V991b was measured after 14 days of incubation, and the growth inhibition rate was calculated. The experimental and calculating method followed the descriptions by Zhang et al. (2015). The experiment was repeated twice.

### Suppression of *Verticillium dahliae* by the Culture Filtrates of the *Fusarium proliferatum* 10R-7

The *F. proliferatum* 10R-7 was inoculated in 100 ml of Czapek–Dox medium in a 250-ml Erlenmeyer flask in shake culture (150 rpm) at 25°C for 6 days. The culture filtrates (CFs) were acquired by centrifuging the culture at 6,000 rpm for 10 min and then filtering through 0.22−μm MS^®^ PES syringe filters. Then, the CFs were added to PDA at a proportion of 1:9 (v/v) with a final CF concentration of 10%. Sterile water was added to PDA as control. Mycelial agar plugs of *V. dahliae* V991b were inoculated on the CF-amended and control PDA. The colony diameter (*CD*) of *V. dahliae* V991b was measured after growing for 14 days at 25°C, and the growth inhibition rate was calculated. The percentage (*P*) of the growth inhibition rate was calculated by the following formula:

(6) $$P\left(\%\right)=\left(CD_{C\,K}-CD_{C\,F}\right)/CD_{C\,K}\times 100$$

where *CD*_*CF*_ represents the colony diameter in the treatment amending with the CFs and *CD*_*CK*_ represents the colony diameter in the control treatment.

### Determination of the Antifungal Activity of the Culture Filtrates of *Fusarium proliferatum* 10R-7

Two different culture media, potato dextrose broth (PDB) medium and Czapek–Dox medium (CDM), were used to incubate isolate 10R-7 for the production of the antifungal metabolites. The ingredients of the PDB medium were as follows: peeled potato, 200 g; glucose, 20 g; and distilled water, 1,000 ml. The ingredients of Czapek–Dox medium were as follows: KNO_3_, 2 g; KH_2_PO_4_, 1 g; MgSO_4_, 0.5 g; KCl, 0.5 g; FeSO_4_, 0.01 g; sucrose, 30 g; and distilled water, 1,000 ml. The isolate 10R-7 was cultured in a PDB or CDM shake culture (150 rpm) at 25°C for 3, 6, 9, 12, 15, and 18 days. The CFs of isolate 10R-7 from each sampling date were obtained and added to PDA at 2, 4, 6, 8, and 10% (v/v), and the PDA without any amendment was treated as control. Mycelial agar plugs of *V. dahliae* were inoculated on the PDA and incubated for 14 days at 25°C. The diameter of each colony was measured and the calculation of the growth inhibition rate was performed as described above.

Moreover, inhibition of spore germination of *V. dahliae* by the CFs of isolate 10R-7 was tested. Plates with water agar (WA) amended with 10% CFs of isolate 10R-7 were prepared. A 20-μl conidial suspension of *V. dahliae* (1 × 10^4^ conidia/ml) was spread evenly on a Petri dish with a glass rod and cultured at 25°C. The control treatment consisted of WA amended with sterile water. The cultures were incubated at 25°C for 6, 12, 24, and 48 h, and at each time point, three dishes for each treatment were sampled and germinated conidia on the media were observed, with 100 conidia per dish. Moreover, 20 germinated conidia on each medium were randomly selected, and the length of the germ tubes was measured.

### Changes in Biomass and pH in the Culture Filtrates of *Fusarium proliferatum* 10R-7

A mycelial agar plug of *F. proliferatum* 10R-7 was shake-inoculated (150 rpm, 25°C) in CDM for 2, 4, 6, 8, and 10 days. At each time point, three cultures were sampled, and the mycelial mass in each culture was collected by filtering, dried at 80°C for 6 h, and weighed. The filtrates were centrifuged at 6,000 rpm at 4°C, and the pH value was measured using a pH meter. The concentration of fusaric acid in the culture filtrates was measured in a spectrophotometer (BioTek, Epoch, Winooski, VT, United States) at 273 nm using pure fusaric acid (Shanghai TCI Development Co., Ltd., Shanghai, China) as reference.

### Identification of the Antifungal Substances Produced by *Fusarium proliferatum* 10R-7

Macroporous resin granules (5 g) were added to 100 ml of culture filtrates (PDB or CDM) of isolate 10R-7, and the mixture was consistently shaken (150 rpm) at 25°C for 10 h. The resin was then washed three times with sterile water and the precipitate was resuspended with 10 ml methanol. The mixture was rotarily evaporated and redissolved in 1 ml of anhydrous ethanol. The solution was loaded in a high-performance liquid chromatography (HPLC) column for semipreparation. The fractions that exhibited antifungal activity against *V. dahliae* were subjected to electrospray ionization mass spectrometric (ESI-MS) analysis (Lyu et al., 2017).

### Antifungal Activity of Fusaric Acid Against *Verticillium dahliae*

Pure fusaric acid (purity: 98%) was purchased from Shanghai TCI Development Co., Ltd. First, 0.01 g of fusaric acid was dissolved in 1 ml of anhydrous ethanol to prepare the mother solution at 10 mg/ml; then, the mother solution was added to PDA to final concentrations of 5, 10, 20, 25, 35, 40, 60, and 80 μg/ml. PDA without amendment of fusaric acid was used as control. There were three replicates for each treatment. *V. dahliae* V991b was inoculated on PDA for different treatments and incubated at 25°C for 7 days. The colony diameter in each dish was measured, and the inhibition rate of fusaric acid on the growth of *V. dahliae* was calculated using the formula mentioned above. The experiment was repeated twice.

### Influence of Fusaric Acid on the Germination of Cotton Seeds and Growth of Cotton Seedlings

The mother solution of 10 mg/ml as mentioned above was diluted with sterile water to prepare solutions of different concentrations of fusaric acid (5, 10, and 20 μg/ml). Cotton seeds (JI11) were surface-sterilized with sodium hypochlorite for 2 min, rinsed with sterile water several times, and then soaked for 30 min in solutions of fusaric acid at different concentrations. Cotton seeds soaked in sterile water for 30 min were used as controls. Then, the seeds were sown in nutrient vermiculite in plastic pots (10 cm × 9 cm, diameter × height) with four seeds in each pot (20 pots for each treatment). The pots were placed in a greenhouse (25 ± 3°C) and watered as required. The number of germinated cotton seeds in each pot was counted after 5 days, and the germination rates were calculated. Then, the seedlings in the pots were thinned to have one seedling per pot. At 20 days postsowing, 20 cotton plants were carefully uprooted and washed under tap water. Growth indices such as stem height, root length, fresh weight, and dry weight (60°C, 48 h) were individually measured. This experiment was performed twice.

### Antifungal Activity of Fusaric Acid on Cotton *Verticillium* Wilt in Greenhouse Experiments

Cotton seedlings were grown in pots to the two-true-leaf stage, and a conidial suspension of *V. dahliae* V991b (5 × 10^6^ conidia/ml) was prepared. The mother solution of fusaric acid was added to a conidial suspension of *V. dahliae* V991b to make the solutions of different concentrations from 2.5 to 80 μg/ml. The experiment was carried out in three trials. Trial 1 included four concentrations of fusaric acid, namely, 2.5, 5, 15, and 25 μg/ml. Trial 2 included three concentrations of fusaric acid, namely, 10, 20, and 40 μg/ml. Trial 3 included three concentrations of fusaric acid, namely, 20, 40, and 80 μg/ml. Then, the roots of cotton seedlings (10 per treatment, three replicates per treatment) were drenched in a conidial suspension of *V. dahliae* V991b (5 × 10^6^ conidia/ml) mixed with fusaric acid for 20 min. Seedlings inoculated with the *V. dahliae* only and sterile water only served as positive and negative controls, respectively. The seedlings were transplanted into nutrition vermiculite in plastic pots (12 cm × 10 cm, diameter × height), with three to four seedlings per pot and three pots per treatment after inoculation. The temperature was maintained at 25 ± 3°C, and the seedlings were watered to maintain an appropriate humidity. The survey method was the same as those mentioned above. The experiment was repeated twice.

### Colonization of *Fusarium proliferatum* 10R-7 on Cotton Roots

The GFP-labeled strain 10R-7GFP was constructed according to *Agrobacterium tumefaciens*-mediated transformation (Zhang et al., 2015). The plasmid camp-GFP containing the GFP gene was provided by Y. J. Huang (School of Life and Medical Sciences, University of Hertfordshire, AL109, Hatfield, AB, United Kingdom).

Isolate 10R-7GFP was derived from the parental isolate 10R-7 and contained a green fluorescent protein gene. Both isolates had similar growth rates on PDA at 25°C (Supplementary Table 2), and isolate 10R-7GFP was stored at −80°C in 20% glycerol (v/v). Pure cultures of *F. proliferatum* 10R-7 and 10R-7GFP were also compared under a microscope (Olympus IX81) equipped with an Olympus U-RFL-T.

To examine the colonization of *F. proliferatum* 10R-7GFP in cotton seedlings, the roots of the two-true-leaf stage cotton seedlings were submerged in a spore suspension of the isolate 10R-7GFP (5 × 10^6^ conidia/ml) for 20 min, and the wild-type isolate 10R-7 and sterile water were used as controls. The seedlings were individually transplanted into nutrition vermiculite in plastic pots (12 cm × 10 cm, diameter × height) at three seedlings per pot, and the temperature was maintained at 25 ± 3°C. Then, the seedlings were sampled at 0, 1, 2, and 7 days postinoculation. The taproots of the samples were cut into slices transversely and longitudinally using a surgical blade. Then, the thin slices were put on the glass slide and observed under a microscope (Olympus IX81) equipped with an Olympus U-RFL-T. The MShot Image Analysis System was used for image analysis.

### Data Analysis

The Statistical Analysis Software (IBM SPSS Statistics 22.0) was used to analyze the data acquired. Significant differences between different treatments were determined using Duncan’s test (*P* < 0.05) *via* one-way ANOVA.

## Results

### Diversity of the Endophytic Fungi in Cotton

One hundred and eighty cotton samples were collected in the test field and 93 isolates of endophytic fungi were isolated from the samples. Twenty species were identified among these isolates according to morphological characteristics and molecular identification (Table 1). The isolated endophytic fungi in cotton were diverse, with Shannon’s diversity (*H*′) and Simpson’s diversity (*D*) indices reaching up to 3.848 and 0.915, respectively. *Fusarium* spp. and *Alternaria* spp. each accounted for 18 of the 93 isolates (19.4% of the total each), and they were the two most dominant genera. *Phomopsis* spp. and *Plectosphaerella cucumerina* each accounted for 10 of 93 isolates. Eight of 93 isolates belonged to *Acrocalymma vagum*, seven isolates to *Diaporthe* spp., five isolates belonged to *Colletotrichum gloeosporioides*, five isolates belonged to *Botryosphaeria dothidea*, three isolates to *Clonostachys rosea*, two isolates belonged to *Leptosphaerulina chartarum*, and two isolates belonged to *Trichoderma* spp. The remaining five fungal species belonged to *Albifimbria viridis*, *Macrophomina phaseolina*, *Phaeosphaeria* spp., *Phoma* spp., and *V. dahliae*. The diversity of endophytic fungi in the roots and stems was different. Among the 93 fungal isolates, 23 were found in the roots and 70 were from the stems. The values for Shannon’s diversity and Simpson’s diversity indices were 2.523 and 0.802 in the roots and 3.593 and 0.895 in the stems, respectively (Table 2). Four species, namely, *A. vagum*, *F. proliferatum*, *Phomopsis* spp., and *P. cucumerina*, were simultaneously isolated from the roots and stems of cotton. Sixteen fungal species were isolated from one of the two cotton tissues.

**TABLE 1:** Identification of the endophytic fungi from cotton by analysis of the ITS (ITS1–5.8S rDNA–ITS2) sequences.

**Isolate**	**Identity to the closest species (GenBank acc. no.)**	**Isolate**	**Identity to the closest species (GenBank acc. no.)**
5R-7	100% to *Acrocalymma vagum* (KF494165)	3S-5	99% to *Fusarium oxysporum* (MG136706)
5R-9	99% to *Acrocalymma vagum* (KP784427)	7S-3	99% to *Fusarium oxysporum* (KF264963)
5S-4	99% to *Acrocalymma vagum* (KP784427)	17S-3	100% to *Fusarium oxysporum* (KU527803)
10R-3	99% to *Acrocalymma vagum* (KX064982)	1S-2	99% to *Fusarium proliferatum* (MF510820)
10R-4	99% to *Acrocalymma vagum* (KX064982)	1S-7	100% to *Fusarium proliferatum* (MF614933)
17R-12	99% to *Acrocalymma vagum* (KX064982)	2S-5	99% to *Fusarium proliferatum* (MF510820)
17R-13	99% to *Acrocalymma vagum* (KX064982)	3S-4	99% to *Fusarium proliferatum* (GQ924905)
17R-14	99% to *Acrocalymma vagum* (KX064987)	10R-2	99% to *Fusarium proliferatum* (MG543724)
6S-6	99% to *Albifimbria viridis* (KU845899)	10R-5	99% to *Fusarium proliferatum* (MF614934)
1S-4	99% to *Alternaria alternata* (KX783388)	10R-7	99% to *Fusarium proliferatum* (MG543724)
1S-8	100% to *Alternaria alternata* (KJ526175)	10R-9	99% to *Fusarium proliferatum* (MG543770)
2S-3	99% to *Alternaria alternata* (KR912224)	10S-7	99% to *Fusarium proliferatum* (FJ648201)
2S-4	99% to *Alternaria alternata* (KX783403)	6R-1	99% to *Fusarium solani* (KX064991)
3S-6	99% to *Alternaria alternata* (KP638335)	7R-2	99% to *Fusarium solani* (KX064991)
3S-7	99% to *Alternaria alternata* (KX858844)	7R-3	99% to *Fusarium solani* (MF800959)
3S-8	99% to *Alternaria alternata* (KY114869)	10R-6	99% to *Fusarium solani* (KU528855)
4S-8	99% to *Alternaria alternata* (KF380821)	17R-8	99% to *Fusarium solani* (MF800959)
4S-18	99% to *Alternaria alternata* (KU982599)	5S-5	99% to *Fusarium nematophilum* (KU324800)
4S-21	99% to *Alternaria alternata* (KU533841)	4S-20	99% to *Leptosphaerulina chartarum* (KJ584553)
4S-22	99% to *Alternaria alternata* (KX783388)	4S-27	99% to *Leptosphaerulina chartarum* (KJ584553)
4S-39	99% to *Alternaria alternata* (KJ526174)	17R-15	99% to *Macrophomina phaseolina* (FJ395243)
4S-40	100% to *Alternaria alternata* (KX783405)	4S-24	99% to *Phaeosphaeria* sp. (HQ914835)
6S-1	99% to *Alternaria alternata* (KF380814)	4S-36	99% to *Phoma* sp. (KT355015)
17S-4	99% to *Alternaria alternata* (KF380821)	1S-6	99% to *Phomopsis* sp. (MF800891)
1S-3	100% to *Alternaria tenuissima* (KU324783)	5S-2	99% to *Phomopsis* sp. (KC172081)
4S-38	99% to *Alternaria tenuissima* (KC337038)	6S-4	99% to *Phomopsis* sp. (KX722227)
10S-1	99% to *Alternaria tenuissima* (KX065003)	7R-4	99% to *Phomopsis* sp. (KC172081)
1S-10	99% to *Botryosphaeria dothidea* (KF293751)	7S-2	99% to *Phomopsis* sp. (KC172081)
5S-7	100% to *Botryosphaeria dothidea* (KR709076)	7S-4	99% to *Phomopsis* sp. (KC172081)
5S-8	100% to *Botryosphaeria dothidea* (KF293759)	7S-5	99% to *Phomopsis* sp. (KC172081)
6S-5	99% to *Botryosphaeria dothidea* (KM433848)	7S-6	99% to *Phomopsis* sp. (KC172081)
9S-1	99% to *Botryosphaeria dothidea* (KF293867)	8S-3	99% to *Phomopsis* sp. (KC172081)
1S-16	99% to *Clonostachys rosea* (MF567519)	8S-4	99% to *Phomopsis* sp. (KC172081)
9S-2	100% to *Clonostachys rosea* (MF663694)	1R-1	99% to *Plectosphaerella cucumerina* (HQ914913)
9S-3	99% to *Clonostachys rosea* (KX783336)	1S-15	99% to *Plectosphaerella cucumerina* (HQ914913)
1S-9	99% to *Colletotrichum gloeosporioides* (KM520010)	4S-14	99% to *Plectosphaerella cucumerina* (HQ914913)
4S-7	99% to *Colletotrichum gloeosporioides* (MF800897)	4S-17	99% to *Plectosphaerella cucumerina* (HQ914913)
4S-9	99% to *Colletotrichum gloeosporioides* (JN887346)	4S-23	99% to *Plectosphaerella cucumerina* (HQ914913)
10S-4	99% to *Colletotrichum gloeosporioides* (JN887348)	5S-3	99% to *Plectosphaerella cucumerina* (HQ914913)
10S-14	99% to *Colletotrichum gloeosporioides* (MF800894)	10R-1	99% to *Plectosphaerella cucumerina* (KC756235)
6S-2	99% to *Diaporthe* sp. (KF928282)	10R-8	99% to *Plectosphaerella cucumerina* (HQ914913)
6S-3	99% to *Diaporthe* sp. (KR709022)	10S-8	99% to *Plectosphaerella cucumerina* (HQ914913)
7S-7	99% to *Diaporthe* sp. (KC172081)	10S-18	99% to *Plectosphaerella cucumerina* (HQ914913)
9S-14	99% to *Diaporthe* sp. (KU324789)	7R-5	99% to *Trichoderma* sp. (KR868247)
17S-1	100% to *Diaporthe* sp. (MF686806)	7R-6	99% to *Trichoderma* sp. (KP689168)
17S-2	99% to *Diaporthe* sp. (MF686806)	5S-10	99% to *Verticillium dahliae* (KY454838)
17S-6	99% to *Diaporthe* sp. (KC172081)		

**TABLE 2:** Endophytic fungi in cotton tissue in Wuhan of central China.

**Species**	**Number of fungal isolates**			**Frequency (%)**
	**Root**	**Stem**	**Total**	
*Acrocalymma vagum*	7	1	8	8.6
*Albifimbria viridis*	NI	1	1	1.1
*Alternaria alternata*	NI	15	15	16.1
*Alternaria tenuissima*	NI	3	3	3.2
*Botryosphaeria dothidea*	NI	5	5	5.4
*Clonostachys rosea*	NI	3	3	3.2
*Colletotrichum gloeosporioides*	NI	5	5	5.4
*Diaporthe* sp.	NI	7	7	7.5
*Fusarium oxysporum*	NI	3	3	3.2
*Fusarium proliferatum*	4	5	9	9.7
*Fusarium solani*	5	NI	5	5.4
*Fusarium nematophilum*	NI	1	1	1.1
*Leptosphaerulina chartarum*	NI	2	2	2.2
*Macrophomina phaseolina*	1	NI	1	1.1
*Phaeosphaeria* sp.	NI	1	1	1.1
*Phoma* sp.	NI	1	1	1.1
*Phomopsis* sp.	1	9	10	10.8
*Plectosphaerella cucumerina*	3	7	10	10.8
*Trichoderma* sp.	2	NI	2	2.2
*Verticillium dahliae*	NI	1	1	1.1
Total number of isolates (*N*)	23	70	93	–
Species richness (*S*)	7	18	20	–
Shannon index of diversity (*H*′)	2.523	3.593	3.848	–
Simpson’s diversity index (*D*)	0.802	0.895	0.915	–

*NI, not isolated.*

### Biocontrol Efficacy in Greenhouse Experiments

**FIGURE 1:**
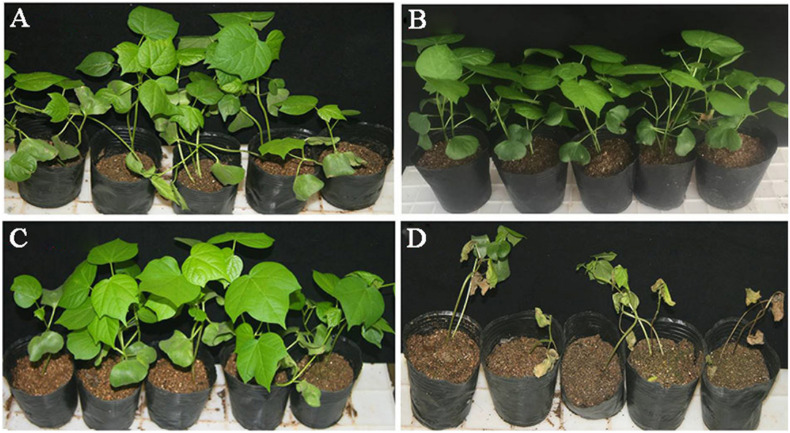
Efficacy of *F. proliferatum* 10R-7 in suppression of *Verticillium* wilt of cotton caused by *Verticillium dahliae* (25 ± 3°C, 30 days postinoculation) in a greenhouse experiment. **(A)** Cotton seedlings treated with sterile water control (CK). **(B)** Cotton seedlings treated with *F. proliferatum* 10R-7 alone (10R-7 alone). **(C)** Cotton seedlings treated with *F. proliferatum* 10R-7 plus *V. dahliae* (10R-7 +*Vd*). **(D)** Cotton seedlings treated with *V. dahliae* alone (*Vd* alone).

Seventy-two spore-producing isolates were tested for the control of *Verticillium* wilt of cotton in the greenhouse. The results are shown in Supplementary Table 1. Five isolates (10R-2, 10R-7, 10R-9, 6R-1, and 17R-8) were obtained through multiple rounds of screening, and the average control efficacy against *Verticillium* wilt was higher than 50% (Table 3). The disease severity and pathogenic incidence were all significantly (*P* < 0.05) reduced relative to those of the pathogen-only control. In trial 1, the biocontrol efficacy of the five isolates 10R-2, 10R-7, 10R-9, 6R-1, and 17R-8 against cotton *Verticillium* wilt ranged from 58.1% to 88.9%. In trial 2, the biocontrol efficacy against cotton *Verticillium* wilt ranged from 44.9% to 80.2%. In trial 3, the biocontrol efficacy against cotton *Verticillium* wilt ranged from 34.4 to 63.4%. The results of the three trials showed that the average biocontrol efficacy of 10R-2, 10R-7, 10R-9, 6R-1, and 17R-8 against cotton *Verticillium* wilt were 59.0, 70.0, 51.8, 58.0, and 65.1%, respectively, and that isolate 10R-7 showed the best inhibitory ability (Figure 1). Among them, isolates 10R-2, 10R-7, and 10R-9 belonged to *F. proliferatum*, and isolates 6R-1 and 17R-8 belonged to *F. solani.*

**TABLE 3:** Control effects of the five antagonists of controlling cotton *Verticillium* wilt in greenhouse.

**Treatment**	**Disease index**	**Biocontrol efficacy (%)**
**Trial 1**		
*F. proliferatum* 10R-2 +*V. dahliae*	34.3 ± 2.2 b^1^	58.1 d^1^
*F. proliferatum* 10R-7 +*V. dahliae*	9.1 ± 1.5 d	88.9 a
*F. proliferatum* 10R-9 +*V. dahliae*	19.6 ± 6.0 c	76.0 c
*F. solani* 6R-1 +*V. dahliae*	14.3 ± 2.4 cd	82.5 b
*F. solani* 17R-8 +*V. dahliae*	16.1 ± 3.6 c	80.4 b
*V. dahliae* alone	81.8 ± 1.5 a	0.0 e
Control (water)	0.0	–
**Trial 2**		
*F. proliferatum* 10R-2 +*V. dahliae*	17.3 ± 2.6 d^1^	80.2 a^1^
*F. proliferatum* 10R-7 +*V. dahliae*	29.2 ± 2.8 c	66.7 b
*F. proliferatum* 10R-9 +*V. dahliae*	48.2 ± 6.0 b	44.9 d
*F. solani* 6R-1 +*V. dahliae*	39.6 ± 8.3 b	54.8 c
*F. solani* 17R-8 +*V. dahliae*	42.4 ± 3.2 b	51.6 c
*V. dahliae* alone	87.5 ± 2.8 a	0.0 e
Control (water)	0.0	–
**Trial 3**		
*F. proliferatum* 10R-2 +*V. dahliae*	45.0 ± 1.1 b^1^	38.6 c^1^
*F. proliferatum* 10R-7 +*V. dahliae*	33.3 ± 6.8 c	54.5 b
*F. proliferatum* 10R-9 +*V. dahliae*	48.1 ± 3.9 b	34.4 c
*F. solani* 6R-1 +*V. dahliae*	46.4 ± 4.8 b	36.6 c
*F. solani* 17R-8 +*V. dahliae*	26.8 ± 2.4 c	63.4 a
*V. dahliae* alone	73.3 ± 4.4 a	0.0 d
Control (water)	0.0	–

*^1^Means ± SE within each column in each trial followed by the same letters are not significantly different (*P* > 0.05) according to Duncan’s multiple test.*

### Suppression Efficacy of the Volatiles and Culture Filtrates of *Fusarium proliferatum* 10R-7

The *F. proliferatum* isolate 10R-7 could produce antifungal substances inhibitory to *V. dahliae*. The inhibition percentages of the volatiles of the isolate 10R-7 were 69.70%. The culture filtrates of isolate 10R-7 were obtained by cultivation of the isolate for 7 days in CDM. The culture filtrates were amended into PDA at the volume ratio of 10% to suppress the growth of *V. dahliae*. The results showed that the inhibition percentage reached up to 100% for the culture filtrates of isolate 10R-7 (Table 4).

**TABLE 4:** Inhibition of mycelial growth of *Verticillium dahliae* by the culture filtrates and the volatiles produced by the *F. proliferatum* 10R-7.

**Isolate**	**Culture filtrates**		**Volatiles**	
	**Colony diameter (cm)**	**Inhibition percentage**	**Colony diameter (cm)**	**Inhibition percentage**
*F. proliferatum* 10R-7	0.0 ± 0.0 b^1^	100.0% a	1.9 ± 0.4 b^1^	69.7% a
Control	5.9 ± 0.1 a	–	6.3 ± 0.4 a	–

*^1^Means ± SE within each column in each trial followed by the same letters are not significantly different (*P* > 0.05) according to Duncan’s multiple test.*

### Control Efficacy of *Fusarium proliferatum* 10R-7 at Different Dosages

The ability of the isolate 10R-7 at four different concentrations to control *V. dahliae* was evaluated in a greenhouse. When the soil was inoculated with a conidial suspension of *F. proliferatum* 10R-7 at 1 × 10^4^, 1 × 10^5^, 1 × 10^6^, or 1 × 10^7^ conidia/ml before inoculation with a spore suspension of *V. dahliae*, a significant difference (*P* < 0.05) in disease index was observed between the treatments of isolate 10R-7 (1 × 10^6^ and 1 × 10^7^ conidia/ml) and the control. The biocontrol efficacy increased with increasing spore concentration for isolate 10R-7. The biocontrol efficacy values for these spore concentrations were 8.7, 16.2, 40.7, and 70.8%, respectively, at 20 dpi, and −3.5, 7.9, 21.1, and 54.4%, respectively, at 25 dpi (Table 5). The efficacy values at 20 and 25 dpi were positively correlated with the spore concentration with correlation coefficients of 0.971 and 0.963, respectively.

**TABLE 5:** Effect of different conidial concentrations of *F. proliferatum* 10R-7 on suppression of cotton *Verticillium* wilt caused by *V. dahliae* in greenhouse.

**Treatment**		**20 days postinoculation**		**25 days postinoculation**	
***F. proliferatum***	***V. dahliae***	**Disease index**	**Biocontrol efficacy (%)**	**Disease index**	**Biocontrol efficacy (%)**
1 × 10^4^ conidia/ml	5 × 10^6^ conidia/ml	80.8 ± 5.2 a^1^	8.7	98.3 ± 2.9 a^1^	−3.5
1 × 10^5^ conidia/ml	5 × 10^6^ conidia/ml	74.2 ± 5.2 a	16.2	87.5 ± 5.0 ab	7.9
1 × 10^6^ conidia/ml	5 × 10^6^ conidia/ml	52.5 ± 10.0 b	40.7	75.0 ± 15.2 b	21.1
1 × 10^7^ conidia/ml	5 × 10^6^ conidia/ml	25.8 ± 9.5 c	70.8	43.3 ± 15.1 c	54.4
0 (water)	5 × 10^6^ conidia/ml	88.5 ± 9.6 a	–	95.0 ± 8.7 ab	–

*^1^Means ± SE within each column in each trial followed by the same letters are not significantly different (*P* > 0.05) according to Duncan’s multiple test.*

### Suppression of *Verticillium dahliae* by the Culture Filtrates of *Fusarium proliferatum* 10R-7

**FIGURE 2:**
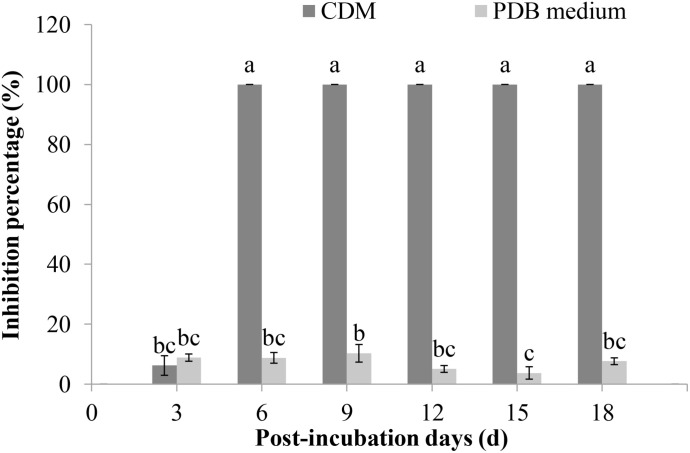
Inhibition rates of *V. dahliae* by the culture filtrates of *F. proliferatum* 10R-7 in CDM (Czapek–Dox medium) and PDB medium at different culture times.

**FIGURE 3:**
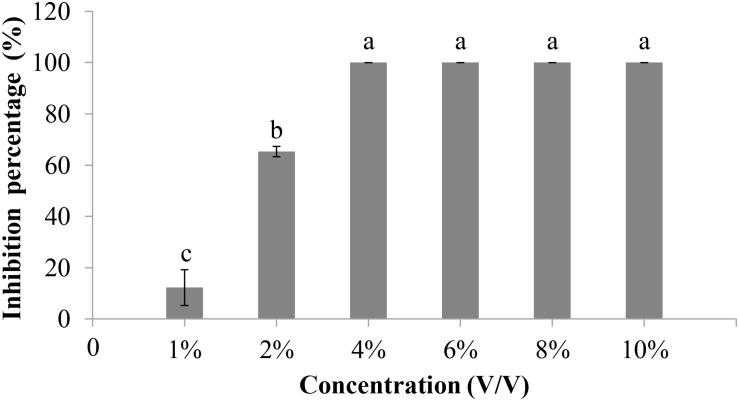
Inhibitory rates of culture filtrates of *F. proliferatum* 10R-7 of different concentrations on *V. dahliae.*

The ability of *F. proliferatum* 10R-7 culture filtrates in PDB and CDM to suppress *V. dahliae* was evaluated. The culture filtrates showed different inhibitory activities. The inhibition percentage by the culture filtrates in CDM was significantly higher (*P* < 0.05) than that of the culture filtrates in PDB. The inhibition percentage was 100% for the 10% 6-day-old CDM culture filtrates and 8.9% for the 6-day-old PDB culture filtrates (Figure 2). The inhibition percentage was 100% for the CDM culture filtrates even at the concentration of 4% (Figure 3).

**FIGURE 4:**
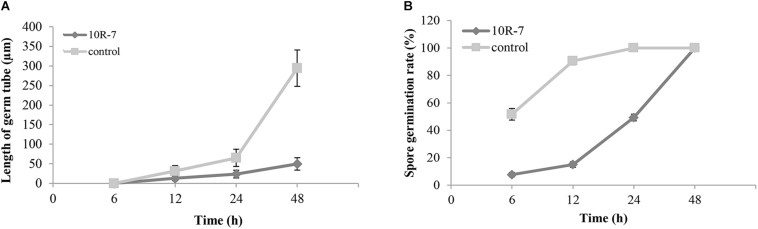
Effect of culture filtrates of *F. proliferatum* 10R-7 (the concentration is 10%) on germ tube elongation **(A)** and spore germination **(B)** of *V. dahliae*.

In the conidial germination test (WA, 25°C), the average spore germination rate of *V. dahliae* was 51.7% at 6 h postincubation (hpi), 90.7% at 12 hpi, and 100% at 24 and 48 hpi in the control treatment without amendment of the cultures of 10R-7 in WA (Figure 4). The average length of the germ tubes was 31.5 μm at 12 hpi and 294.3 μm at 48 hpi. When the culture filtrates of 10R-7 were amended in WA (10%), however, the percentages of germinated conidia were reduced to 7.7, 15.0, and 49.3% at 6, 12, and 24 hpi, respectively, and at 48 hpi, the value reached up to 100.0%. The average length of the germ tubes was reduced to 13.0, 23.8, and 49.8 μm, at 12, 24, and 48 hpi, respectively (Figure 4). The results indicated that the culture filtrates of isolate 10R-7 had inhibitory effects both on spore germination and on elongation of germ tubes of *V. dahliae*.

### Time Course of Biomass, pH, and Content of Fusaric Acid in the Cultures of *Fusarium proliferatum* 10R-7

The biomass of *F. proliferatum* 10R-7 in CDM cultures (25°C) increased with time, reached the maximum value at 8 dpi, and had a small decrease at 10 dpi. The biomass rapidly increased threefold from 2 to 4 dpi. The pH value increased from 7.3 to 8.4 from 2 to 10 dpi.

**FIGURE 5:**
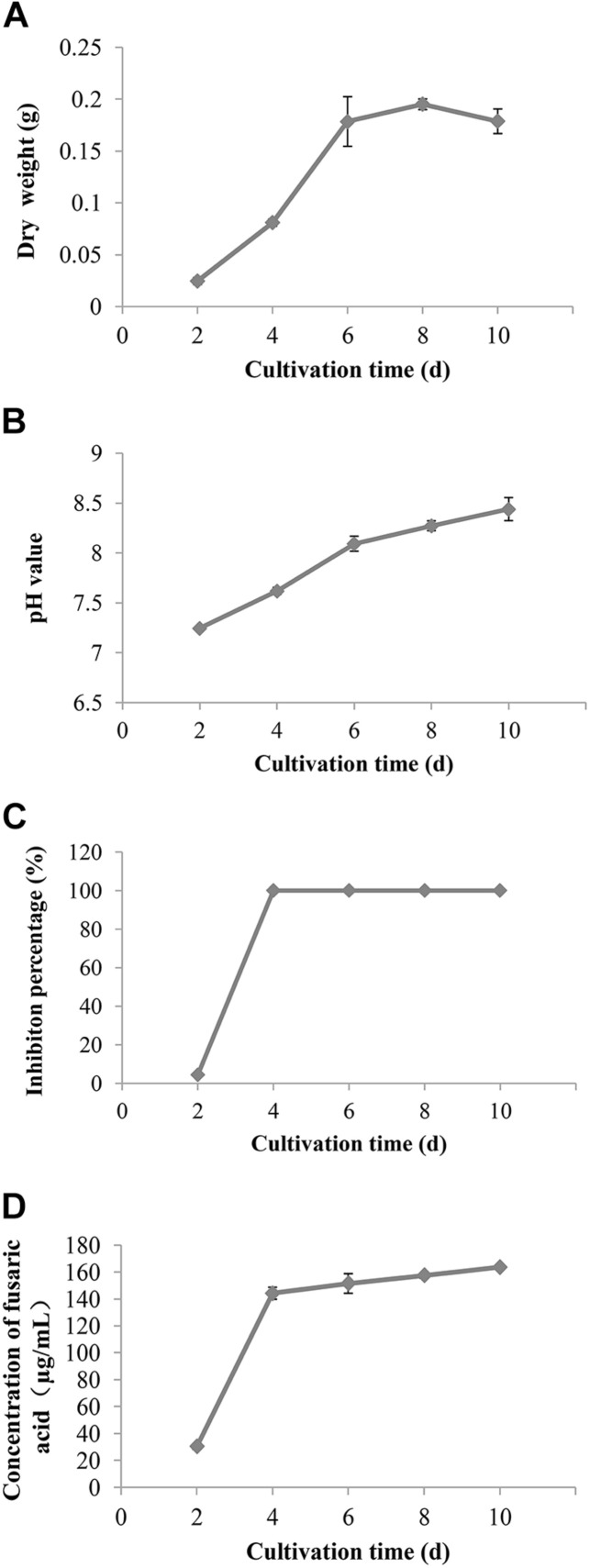
The change of the biomass **(A)**, pH value **(B)**, inhibition rate **(C)**, and content of fusaric acid **(D)** of the culture filtrates of *F. proliferatum* 10R-7 on *V. dahliae* at different culture times.

The inhibition percentages against *V. dahliae* reached 100% at 4 dpi. At the same time, the absorption values of the culture filtrates at 273 nm at different cultivation times were measured to calculate the fusaric acid content. The fusaric acid content increased rapidly when the cultivation time increased from 2 to 4 dpi and increased slowly when the cultivation time further increased from 4 to 10 dpi (Figure 5). These results suggest that the fusaric acid content change was consistent with the change in the inhibition activity of the culture filtrates against *V. dahliae*.

### Identification of the Antifungal Substances Produced by *Fusarium proliferatum* 10R-7

High-performance liquid chromatography and ESI-MS analyses were applied to analyze the antifungal substances in the culture filtrates of *F. proliferatum* 10R-7. ESI-MS (3.5 kV) showed a molecular ion peak at *m*/*z* 180.02 (M+H)^+^ and 178.2 (M-H)^–^, and the ESI-MS molecular weight was 179.2 Da. Based on these data, the molecular formula of the antifungal substance was inferred to be C_10_H_13_NO_2_. The UV maximum absorbance was at 270.9 nm. We detected fusaric acid in the culture filtrates of *F. proliferatum* 10R-7 using HPLC and ESI-MS analysis. The HPLC chromatography, TIC, and the UV absorption results of fusaric acid were similar with those of the culture filtrates (Supplementary Figure 1). It was therefore inferred that the antifungal substance in the culture filtrates is fusaric acid. However, the culture filtrates in CDM gave UV maximum absorbance at 272.8 nm, while the culture filtrates in PDB gave UV maximum absorbance at 309.8 nm (Supplementary Figure 2). This result showed that the secondary metabolites of *F. proliferatum* 10R-7 in CDM and PDB differed.

### Antifungal Activity of Fusaric Acid

**FIGURE 6:**
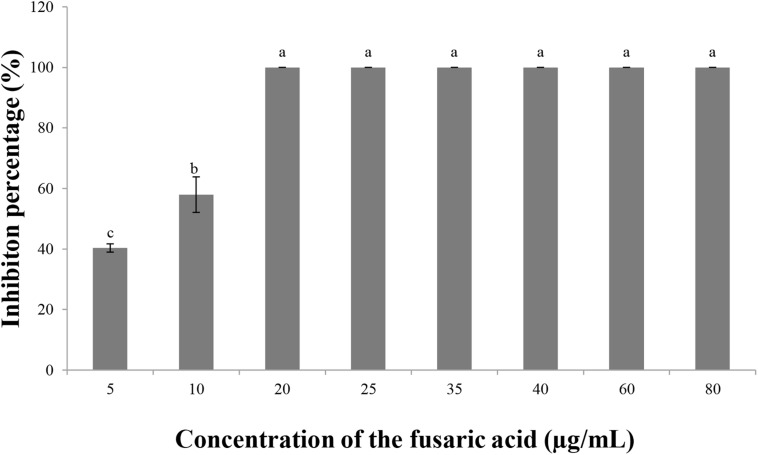
Inhibitory effect of fusaric acid of different concentrations on *V. dahliae.*

Fusaric acid solution suppressed the growth of *V. dahliae*. The inhibition percentages at 5 and 10 μg/ml were 40.3 and 57.9%, respectively, and the inhibition percentage reached 100% when the concentration was 20 μg/ml or higher (Figure 6). These results also verified that fusaric acid may be the antifungal substance in the culture filtrates of *F. proliferatum* 10R-7.

### Influence of Fusaric Acid Concentrations on the Growth of Cotton Seedlings

High concentrations of fusaric acid had a negative effect on the growth of cotton seedlings. When the concentration was reduced, the negative effect decreased. We surveyed the growth index of the cotton seedlings after treatments with different concentrations (5, 10, and 20 μg/ml). The results showed that fusaric acid at 5 μg/ml did not produce a negative effect on the growth of the cotton seedlings. When the concentration was increased to 10 or 20 μg/ml, a negative effect on the growth of seedlings as measured by stem height, fresh weight, and dry weight was observed (Table 6).

**TABLE 6:** Effects of the different concentrations of fusaric acid on growth index of cotton.

**Treatment**	**Stem height (cm)**	**Root length (cm)**	**Fresh weight (g)**	**Dry weight (g)**
Control	15.2 ± 2.2 a^1^	9.1 ± 2.3 a^1^	2.5 ± 0.7 a^1^	0.2 ± 0.1 a^1^
Fusaric acid (5 μg/ml)	14.2 ± 1.7 ab	9.9 ± 2.8 a	2.4 ± 0.4 a	0.2 ± 0.1 a
Fusaric acid (10 μg/ml)	13.6 ± 1.9 b	8.8 ± 1.6 a	2.0 ± 0.4 b	0.2 ± 0.0 b
Fusaric acid (20 μg/ml)	13.4 ± 1.2 b	7.1 ± 2.6 b	2.0 ± 0.6 b	0.2 ± 0.1 ab

*^1^Means ± SE within each column in each trial followed by the same letters are not significantly different (*P* > 0.05) according to Duncan’s multiple test.*

### Influence of Fusaric Acid Concentrations on Cotton *Verticillium* Wilt

The culture filtrates of isolate 10R-7 exhibited antifungal activity against *V. dahliae*, and the antifungal substance was deduced to be fusaric acid. We measured the ability of fusaric acid to control cotton *Verticillium* wilt in a greenhouse experiment. Fusaric acid was applied at different concentrations (2.5, 5, 10, 15, 20, 25, 40, and 80 μg/ml). Fusaric acid did not significantly (*P* > 0.05) reduce the severity of the disease (Table 7). It may play a minor role in the suppression of the disease caused by *V. dahliae*.

**TABLE 7:** Effects of the different concentrations of fusaric acid on cotton *Verticillium* wilt in greenhouse experiment.

**Treatment**	**Disease incidence**	**Disease index**
**Trial 1**		
*V. dahliae* alone	86.1% a^1^	66.7 ± 15.7 a^1^
2.5 μg/ml fusaric acid + *V. dahliae*	77.0% a	56.9 ± 22.9 a
5 μg/ml fusaric acid + *V. dahliae*	77.8% a	61.9 ± 8.6 a
15 μg/ml fusaric acid + *V. dahliae*	72.2% a	57.6 ± 10.7 a
25 μg/ml fusaric acid + *V. dahliae*	65.8% a	50.6 ± 26.0 a
**Trial 2**		
*V. dahliae* alone	100.0% a^1^	90.0 ± 5.0 a
10 μg/ml fusaric acid + *V. dahliae*	86.7% b	80.8 ± 19.1 a
20 μg/ml fusaric acid + *V. dahliae*	96.7% ab	74.2 ± 12.3 a
40 μg/ml fusaric acid + *V. dahliae*	100.0% a	83.3 ± 2.9 a
**Trial 3**		
*V. dahliae* alone	97.8% a^1^	73.3 ± 11.6 a
20 μg/ml fusaric acid + *V. dahliae*	78.9% ab	56.4 ± 11.0 a
40 μg/ml fusaric acid + *V. dahliae*	77.1% ab	51.9 ± 13.8 a
80 μg/ml fusaric acid + *V. dahliae*	68.9% b	46.1 ± 21.8 a

*^1^Means ± SE within each column in each trial followed by the same letters are not significantly different (*P* > 0.05) according to Duncan’s multiple test.*

### Colonization of *Fusarium proliferatum* 10R-7 on Cotton Roots

**FIGURE 7:**
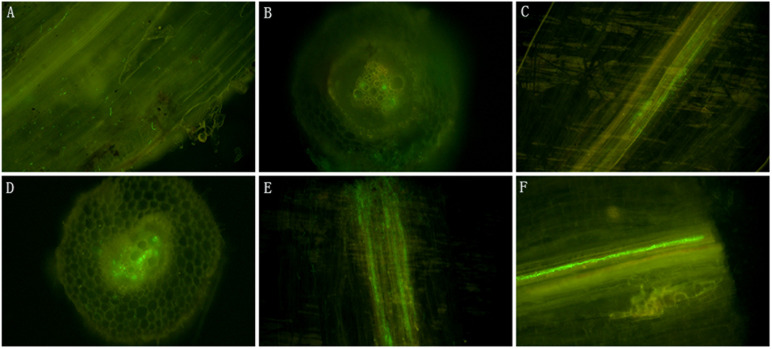
The microscopic micrographs showing endophytic colonization of cotton roots by *Fusarium proliferatum* 10R-7GFP. **(A)** Longitudinal sections of the roots inoculated with *F. proliferatum* 10R-7GFP (25°C, half past hour postinoculation); **(B,C)** longitudinal and cross sections of the roots inoculated with *F. proliferatum* 10R-7GFP at the first day postinoculation, respectively; **(D,E)** longitudinal and cross sections of the roots inoculated with *F. proliferatum* 10R-7GFP at the second day postinoculation, respectively. **(F)** Longitudinal sections of the roots inoculated with *F. proliferatum* 10R-7GFP at the seventh day postinoculation.

In contrast to the wild-type isolate 10R-7, isolate 10R-7GFP emitted green fluorescence in hyphae and conidia (Supplementary Figure 3). The 10R-7GFP-treated cotton seedlings were sampled and the taproots were observed under an Olympus IX81 for endophytic colonization by 10R-7GFP. While green fluorescence was not detected in the control group treated with water and the wild-type isolate 10R-7, green fluorescence was detected in the cotton root group inoculated with isolate 10R-7GFP. The conidia of 10R-7GFP attached to the surface of the cotton roots half an hour after inoculation with isolate 10R-7GFP. The conidia germinated, formed hyphae, and entered into the taproots at 1, 2, and 7 dpi. The hyphae of 10R-7GFP were observed in the intercellular space and vascular bundles of the taproots of cotton seedlings at 1 dpi. At 7 dpi, the hyphae had colonized mainly along the axis of the taproots of cotton seedlings (Figure 7).

## Discussion

In the present research, 93 fungal isolates were obtained from surface-sterilized tissues of cotton grown in Hubei Province of central China. The results suggested that the endophytic fungi residing in cotton plants are highly diverse. These isolates were identified as belonging to 20 species according to morphological characteristics and ITS sequence identification, including *Fusarium* spp., *Alternaria* spp., *Phomopsis* spp., *P. cucumerina*, *A. vagum*, *Diaporthe* spp., *Colletotrichum* spp., *B. dothidea*, *Bionectria ochroleuca*, *L. chartarum*, *Trichoderma* spp., and others. Among these species, the most dominant were *Fusarium* spp. and *Alternaria* spp. Previous research reported 373 isolates from the endorhiza, rhizosphere, and bulk soil of cotton plants, and 12 fungal antagonists against *V. dahliae* were obtained (Zheng et al., 2011). Among these 12 fungal antagonists screened, five belonged to *Fusarium* spp., accounting for almost half of the 12 isolates. The results also showed that *Fusarium* spp. may be a dominant species of cotton endophytes. The community diversity of the endophytic fungi in cotton in this study was not very comprehensive due to the limitation of the samples, unculturable endophytic fungi, etc. Additional studies using extensive sampling strategies such as molecular sequencing technology are necessary to obtain more complete data for the endophytic fungal community in cotton. In addition to studying the diversity of the endophytes in cotton, our other main objective was to screen potential BCAs against cotton *Verticillium* wilt.

Biological control has attracted increasing attention from researchers for suppressing soilborne diseases such as *Verticillium* wilt because of its environmental friendliness and pollution avoidance due to reduced pesticide use. However, the first step is to find a suitable source of potential BCAs. Previous studies indicated that endophytes were a rich source of BCAs. Huang et al. (2020) evaluated the capacity of endophytic fungi isolated from cucurbit plants to control soilborne diseases, and the results showed that endophytic fungi from cucurbits have great potential as biocontrol agents. The endophyte *Cladorrhinum foecundissimum* could colonize the roots of cotton to promote the absorption of phosphorus and plant growth (Gasoni and Stegman de Gurfinkel, 1997). Li et al. (2014) reported that a total of 642 endophytic fungi from 27 genera were isolated from the roots (141), stems (111), and leaves (391) of *G. hirsutum* plants. Thirty-nine isolates showed different degrees of antifungal activity against *V. dahliae* VD080. Narisawa et al. (2002) reported that a few fungal endophytes from eggplants, melon, tomato, strawberry, and Chinese cabbage are potential BCAs for *Verticillium* wilt of eggplant.

Moreover, the screening strategy is crucial. The traditional screening strategy is to evaluate the *in vitro* antagonism of screening strains toward *V. dahliae* in the first round of screening. However, some of the strains might be effective in controlling cotton *Verticillium* wilt while lacking *in vitro* antagonism. Therefore, we adopted the strategy of using greenhouse experiments for the first round of screening. First, we discarded those of the 93 isolates that did not produce spores. Then, the remaining 72 isolates were further screened according to screening in greenhouse experiments. The results revealed some potential biocontrol strains among these cotton endophytes. Five fungal antagonists were obtained according to multiple screening, and their average biocontrol efficiencies against cotton *Verticillium* wilt in the greenhouse experiment were higher than 50%. The five most effective fungal antagonists screened in greenhouse experiments all belong to *Fusarium* spp. One possible reason is that the environment (temperature and soil) is suitable for *Fusarium* spp. to grow in central China and *Fusarium* spp. is a dominant genera in isolated endophytic fungi. The other possible reason is that it is related to the screening method of greenhouse experiment used in research. In the screening experiment, the conidial suspension of *V. dahliae* mixed with endophytic fungi was inoculated with the cotton seedlings and *Fusarium* spp. have faster growth rate and stronger competitive ability than *V. dahliae*. Numerous studies have reported that nonpathogenic isolates of *F. oxysporum* have the potential to protect plants against pathogenic *F. oxysporum* and *V. dahliae* through competition for nutrients and space, induction of resistance responses, and production of antibiotic substances (Fravel et al., 2003; Pantelides et al., 2009; Zhang et al., 2015). These endophytes will be screened to be potential BCAs to suppress cotton *Verticillium* wilt in the future.

The most effective isolate 10R-7 screened in this study produced volatile organic compounds (VOCs) and secondary metabolism products that inhibit the growth of *V. dahliae* on agar media. The results showed that compared with the control treatment, *F. proliferatum* 10R-7 treatment significantly reduced wilt symptom development in cotton under greenhouse conditions. This is the first report about the biocontrol efficacy of the *F. proliferatum* against *V. dahliae*. Then, the biocontrol mechanisms of *F. proliferatum* 10R-7 were investigated in terms of competition for ecological sites and production of antibiotic substances. The inhibition percentage of VOCs that *F. proliferatum* 10R-7 produced was 69.71%, and previous research reported that an endophyte *F. oxysporum* CanR-46 from oilseed rape produced VOCs that suppressed the growth of pathogens *Botrytis cinerea* and *Sclerotinia sclerotiorum*. GC-MS analysis identified a total of 19 main VOCs. Whether the VOCs that *F. proliferatum* 10R-7 produced were similar to the VOCs of *F. oxysporum* CanR-46 will be studied in the future.

The CFs of *F. proliferatum* 10R-7 shake-cultured in CDM for 7 days inhibited mycelial growth, spore germination, and mycelial elongation of *V. dahliae*. The CFs inhibited *V. dahliae* growth, and the inhibition rate of 4% CFs reached 100% on agar media. Analysis of the CFs of *F. proliferatum* 10R-7 shake-cultured in CDM showed that the main antifungal substance was fusaric acid, and the results showed that fusaric acid also inhibited *V. dahliae* growth. The inhibition rate of 20 μg/ml fusaric acid reached 100% on agar media. However, as a type of *Fusarium* toxin, fusaric acid had a negative impact on plant growth and caused the wilting of plants at high concentrations. It was confirmed that 10 and 20 μg/ml fusaric acid had negative impacts on plant growth. However, when cotton roots were inoculated with *F. proliferatum* 10R-7, fusaric acid was not produced in the root exudates (data not shown). Fusaric acid was also not produced when *F. proliferatum* 10R-7 was shake-cultured in PDB medium. The results indicated that *F. proliferatum* 10R-7 produced fusaric acid under certain conditions. A high concentration of fusaric acid could produce a negative effect and cause the wilting of plants, and concentrations of fusaric acid below a certain level did not cause inhibition. Therefore, pure fusaric acid did not play a good biocontrol role. When a spore suspension of *V. dahliae* mixed with different concentrations of fusaric acid was inoculated, the development of *Verticillium* wilt symptoms in cotton did not significantly decrease. It seemed that the production of fusaric acid had a minor correlation with the biocontrol effect of *F. proliferatum* 10R-7 against *V. dahliae.* The production of fusaric acid was verified to not be the main factor in the biocontrol mechanism of *F. proliferatum* 10R-7. Therefore, we analyzed another category: competition for space.

For this purpose, we transformed *F. proliferatum* 10R-7 with a GFP gene. GFP transformation has become a common method for detection in plant–pathogen interaction studies. GFP-labeled *F. proliferatum* 10R-7 showed the same characteristics as the wild-type strain in morphology, growth rates, and control effects for *V. dahliae* in the greenhouse experiments (Supplementary Tables 2, 3 and Supplementary Figure 3). Previous research has shown dynamic changes in *V. dahliae* at the root after the cotton roots had been inoculated. At 1 dpi, the conidia on root surfaces germinated and formed hyphal networks along the cell junctions (Pantelides et al., 2009; Zhao et al., 2014). By approximately 7 dpi, *V. dahliae* had penetrated and massively colonized the xylem vessels of the roots. It seems that *F. proliferatum* 10R-7 entered the root and dominated the same ecological niche when the roots were inoculated with the spore suspension of GFP-labeled *F. proliferatum* 10R-7. This could result from competition for space, one of the main mechanisms of action of BCAs against pathogens (Pantelides et al., 2009; Gizi et al., 2011). Meanwhile, when the cotton roots were inoculated with different concentrations (1 × 10^4^, 1 × 10^5^, 1 × 10^6^, and 1 × 10^7^ conidia/ml) of *F. proliferatum* 10R-7 spore solutions before being inoculated with a spore suspension of *V. dahliae*, the biocontrol efficacy increased as the spore concentration increased, and the biocontrol efficacy of 1 × 10^7^ conidia/ml of *F. proliferatum* 10R-7 spore solutions was greater than those of the other treatments. This result also indicated that when the inoculation spore concentrations increased, *F. proliferatum* 10R-7 dominated the ecological niche to a greater degree to prevent the invasion of the pathogen *V. dahliae*.

Analysis of the biocontrol mechanism of BCAs will help us to improve control strategies. Another important problem is the safety of BCAs. In the present study, two inoculation methods, the mixed inoculation with a spore suspension of *F. proliferatum* 10R-7 and *V. dahliae* and the inoculation with a spore suspension of *F. proliferatum* 10R-7 before inoculation with *V. dahliae*, could both reduce the symptom development of *Verticillium* wilt in cotton significantly in greenhouse experiments. However, the toxic substance fusaric acid could be produced in the CFs of *F. proliferatum* 10R-7 shake-cultured in Czapek–Dox medium. The production of the toxin means that the application of *F. proliferatum* 10R-7 poses some risk to some other plants. Therefore, future studies will focus on how to avoid the negative effect when taking full advantage of BCAs.

Overall, we investigated two possible mechanisms of action of *F. proliferatum* 10R-7 against *V. dahliae*: the production of antifungal substances and the competition for space. Some previous research has revealed the biocontrol mechanism of nonpathogenic *F. oxysporum* against *V. dahliae*. However, to our knowledge, this is the first study on the biocontrol mechanism of *F. proliferatum* against *V. dahliae*. Our results showed that competition for space on the root surface may be the main biocontrol mechanism that *F. proliferatum* 10R-7 uses against *V. dahliae*. The production of secondary metabolites was not considered to be the biocontrol mechanisms of *F. proliferatum* 10R-7 against *V. dahliae*. Promoting the application of *F. proliferatum* 10R-7 as a promising BCA warrants further study.

## Conclusion

Ninety-three endophytic fungal strains were isolated from healthy cotton samples: 23 strains from the roots and 70 strains from the stems. These strains were identified as belonging to 20 species according to morphological characterization and ITS sequence analysis. *Fusarium* and *Alternaria* were the two dominant genera, constituting 19.4% of the total strains. The diversity of the cotton endophytes was analyzed. Then, 72 spore-producing strains were tested for the suppression of CVW caused by *V. dahliae* in a greenhouse. Five strains exhibited effective suppression of CVW with average efficacy values higher than 50%. As one of the effective strains, the possible biocontrol mechanisms of *F. proliferatum* 10R-7 against *V. dahliae* were investigated by identifying the antifungal metabolites of *F. proliferatum* 10R-7 and assessing its colonization in cotton roots. The results showed that endophytic colonization may play a role in the suppression of infection of cotton by *V. dahliae.*

## Data Availability Statement

The original contributions presented in the study are included in the article/Supplementary Material, further inquiries can be directed to the corresponding author/s.

## Author Contributions

LJ, LY, GL, and PW conceived and designed the experiments. LJ, WL, and DX performed the experiments. LJ and NY organized and analyzed the data. LJ, PW, and GL wrote the manuscript. GL supervised the study. All authors read and approved the final manuscript.

## Conflict of Interest

The authors declare that the research was conducted in the absence of any commercial or financial relationships that could be construed as a potential conflict of interest.

## Publisher’s Note

All claims expressed in this article are solely those of the authors and do not necessarily represent those of their affiliated organizations, or those of the publisher, the editors and the reviewers. Any product that may be evaluated in this article, or claim that may be made by its manufacturer, is not guaranteed or endorsed by the publisher.

## Supplementary Material

Supplementary Figure 1 HPLC and TIC comparison and analysis of the culture filtrates of *F. proliferatum* 10R-7 and fusaric acid. **(A)** HPLC chromatography and TIC (total ion chromatogram) of the culture filtrates of *F. proliferatum* 10R-7. **(B)** HPLC chromatography and TIC of fusaric acid. **(C)** UV absorption spectrogram of the culture filtrates of *F. proliferatum* 10R-7. **(D)** UV absorption spectrogram of fusaric acid.

Supplementary Figure 2 TIC and UV absorption spectrogram of the culture filtrates of *F. proliferatum* 10R-7 in PDB medium and CDM (Czapek-Dox medium). **(A)** TIC of the culture filtrates of *F. proliferatum* 10R-7 in PDB medium. **(B)** UV absorption spectrogram of the culture filtrates of *F. proliferatum* 10R-7 in PDB medium. **(C)** TIC of the culture filtrates of *F. proliferatum* 10R-7 in CDM. **(D)** UV absorption spectrogram of the culture filtrates of *F. proliferatum* 10R-7 in CDM.

Supplementary Figure 3 The morphological characteristics of *F. proliferatum* 10R-7 and *F. proliferatum* 10R-7GFP. **(A,B)** The colony morphology of *F. proliferatum* 10R-7 and *F. proliferatum* 10R-7GFP, respectively. **(C,D)** Mycelial and conidial morphology of the proliferatum 10R-7GFP.

Supplementary Table 1 The preliminary screening of the endophytes against *Verticillium dahliae.*

Supplementary Table 2 The comparison of growth rates of the two isolates *F. proliferatum* 10R-7 and *F. proliferatum* 10R-7GFP.

Supplementary Table 3 Control effect of *F. proliferatum* 10R-7GFP on suppression of cotton Verticillium wilt caused by *V. dahliae* in greenhouse.
