# Supplementary material for: Diversity and Biocontrol Potential of Culturable Endophytic Fungi in Cotton
Source: Front Microbiol. 2021 Aug 13;12:698930. doi: 10.3389/fmicb.2021.698930 (PMC8415002; doi:10.3389/fmicb.2021.698930)
Supplement: Supplementary Figure 1 — HPLC and TIC comparison and analysis of the culture filtrates of F. proliferatum 10R-7 and fusaric acid. (A) HPLC chromatography and TIC (total ion chromatogram) of the culture filtrates of F. proliferatum 10R-7. (B) HPLC chromatography and TIC of fusaric acid. (C) UV absorption spectrogram of the culture filtrates of F. proliferatum 10R-7. (D) UV absorption spectrogram of fusaric acid. [file Data_Sheet_1.docx]

Table S1. The preliminary screening of the endophytes against *Verticillium dahliae*

| Isolate | Control efficiency（%） |
| --- | --- |
| *Alternaria alternata* 1S-4 | -18.1 |
| *Alternaria alternata* 1S-8 | 59.3 |
| *Alternaria alternata* 2S-3 | -13.4 |
| *Alternaria alternata* 2S-4 | 15.6 |
| *Alternaria alternata* 3S-6 | 12.2 |
| *Alternaria alternata* 3S-7 | 0.7 |
| *Alternaria alternata* 3S-8 | -8.7 |
| *Alternaria alternata* 4S-18 | 10.2 |
| *Alternaria alternata* 4S-21 | -30.2 |
| *Alternaria alternata* 4S-22 | 15.0 |
| *Alternaria alternata* 4S-39 | 12.2 |
| *Alternaria alternata* 4S-40 | -14.6 |
| *Alternaria alternata* 4S-8 | -17.5 |
| *Alternaria alternata* 6S-1 | 47.0 |
| *Alternaria alternata* 17S-4 | 14.4 |
| *Alternaria tenuissima* 4S-38 | -9.2 |
| *Alternaria tenuissima* 10S-1 | 43.1 |
| *Acrocalymma vagum* 5R-7 | 8.3 |
| *Acrocalymma vagum* 5R-9 | 76.6 |
| *Acrocalymma vagum* 10R-3 | -11.4 |
| *Acrocalymma vagum* 10R-4 | -22.8 |
| *Acrocalymma vagum* 17R-12 | -22.8 |
| *Acrocalymma vagum* 17R-13 | 30.8 |
| *Acrocalymma vagum* 17R-14 | 10.2 |
| *Acrocalymma vagum* 5S-4 | -8.7 |
| *Botryosphaeria dothidea* 5S-8 | 33.0 |
| *Botryosphaeria dothidea* 6S-5 | 13.1 |
| *Clonostachys rosea* 9S-2 | -15.3 |
| *Clonostachys rosea* 9S-3 | -8.1 |
| *Colletotrichum gloeosporioides* 1S-9 | -5.5 |
| *Colletotrichum gloeosporioides* 4S-7 | 9.6 |
| *Colletotrichum gloeosporioides* 4S-9 | 25.6 |
| *Colletotrichum gloeosporioides* 10S-4 | 26.8 |
| *Colletotrichum gloeosporioides*10S-14 | 14.3 |
| *Diaporthe* sp.6S-2 | 7.9 |
| *Diaporthe* sp.6S-3 | 11.9 |
| *Diaporthe* sp.7S-7 | -3.8 |
| *Diaporthe* sp.9S-14 | -13.7 |
| *Fusarium oxysporum* 3S-5 | -11.6 |
| *Fusarium oxysporum* 5S-5 | 28.7 |
| *Fusarium oxysporum* 7S-3 | 31.3 |
| *Fusarium oxysporum* 17S-3 | 69.4 |
| *Fusarium proliferatum* 10R-2 | 58.1 |
| *Fusarium proliferatum* 10R-5 | 40.9 |
| *Fusarium proliferatum* 10R-7 | 88.89 |
| *Fusarium proliferatum* 10R-9 | 76.00 |
| *Fusarium proliferatum* 1S-2 | 30.8 |
| *Fusarium proliferatum* 1S-7 | 27.4 |
| *Fusarium proliferatum* 2S-5 | 37.1 |
| *Fusarium proliferatum* 3S-4 | 30.3 |
| *Fusarium proliferatum* 10S-7 | 59.3 |
| *Fusarium solani* 6R-1 | 82.5 |
| *Fusarium solani* 7R-2 | 53.0 |
| *Fusarium solani* 7R-3 | 57.5 |
| *Fusarium solani* 10R-6 | 44.7 |
| *Fusarium solani* 17R-8 | 80.4 |
| *Leptosphaerulina chartarum* 4S-27 | 15.0 |
| *Macrophomina phaseolina* 17R-15 | 69.4 |
| *Phaeosphaeria* sp. 4S-24 | 10.0 |
| *Phomopsis* sp. 8S-4 | 3.8 |
| *Plectosphaerella cucumerina* 1R-1 | 14.8 |
| *Plectosphaerella cucumerina* 10R-1 | 79.9 |
| *Plectosphaerella cucumerina* 10R-8 | 26.8 |
| *Plectosphaerella cucumerina* 1S-15 | 52.1 |
| *Plectosphaerella cucumerina* 4S-14 | 45.0 |
| *Plectosphaerella cucumerina* 4S-17 | -20.5 |
| *Plectosphaerella cucumerina* 4S-23 | 11.4 |
| *Plectosphaerella cucumerina* 5S-3 | 73.4 |
| *Plectosphaerella cucumerina* 10S-8 | 37.9 |
| *Plectosphaerella cucumerina* 10S-18 | 7.5 |
| *Trichoderma* sp. 7R-6 | -29.9 |
| *Verticillium dahliae* 5S-10 | 5.1 |

Table S2. The comparison of growth rates of the two isolate *F. proliferatum* 10R-7 and *F. proliferatum* 10R-7GFP

| Treatment | Colony diameter (cm) after 7 days cultivation |
| --- | --- |
| *F. proliferatum* 10R-7 | 6.4±0.3a^1^ |
| *F. proliferatum* 10R-7GFP | 6.3±0.1a |

1 Means ± SE within each column in each trial followed by the same letters are not significantly different (P > 0.05) according to Duncan’s multiple test.

Table S3. Control effect of *F. proliferatum* 10R-7GFP on suppression of cotton Verticillium wilt caused by *V. dahliae* in greenhouse.

| Treatment | 25 days post-inoculation | | 30 days post-inoculation | |
| --- | --- | --- | --- | --- |
|  | Disease index | Biocontrol efficacy (%) | Disease index | Biocontrol efficacy (%) |
| *F. proliferatum* 10R-7GFP | 24.1±1.6b^1^ | 40.3 | 35.8±5.1b^1^ | 47.2 |
| Control | 40.3±2.6a | - | 67.8±4.1a | - |

1 Means ± SE within each column in each trial followed by the same letters are not significantly different (P > 0.05) according to Duncan’s multiple test.


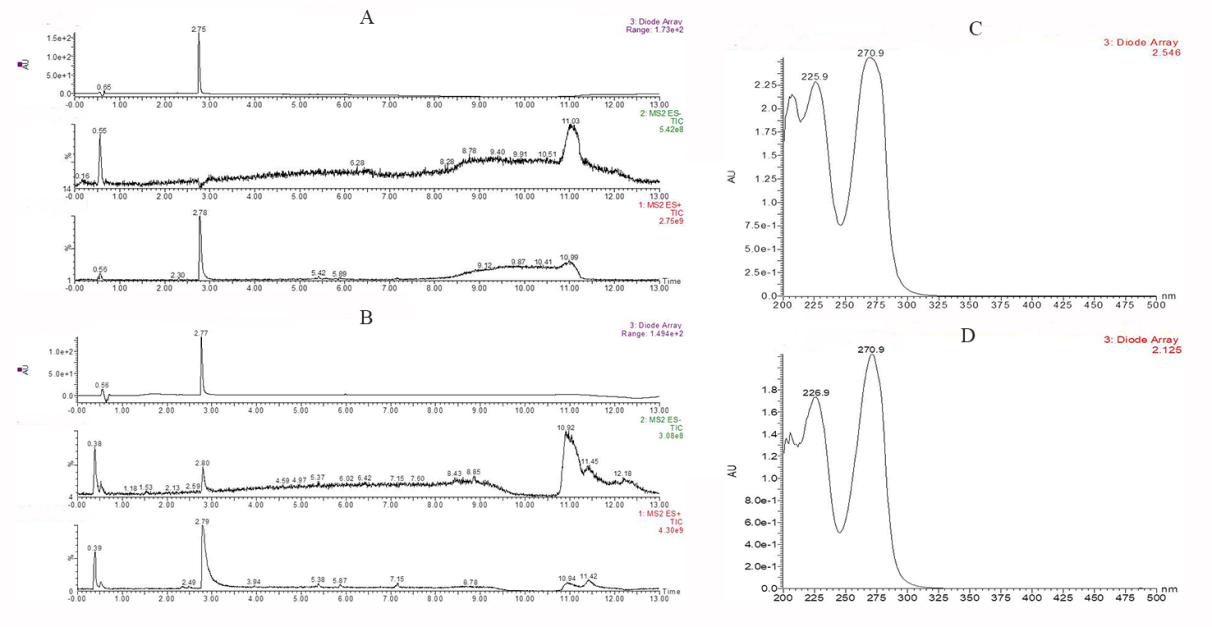


FIG S1 HPLC and TIC comparison and analysis of the culture filtrates of *F. proliferatum* 10R-7 and fusaric acid. A: HPLC chromatography and TIC (total ion chromatogram) of the culture filtrates of *F. proliferatum* 10R-7. B: HPLC chromatography and TIC of fusaric acid. C: UV absorption spectrogram of the culture filtrates of *F. proliferatum* 10R-7. D: UV absorption spectrogram of fusaric acid.


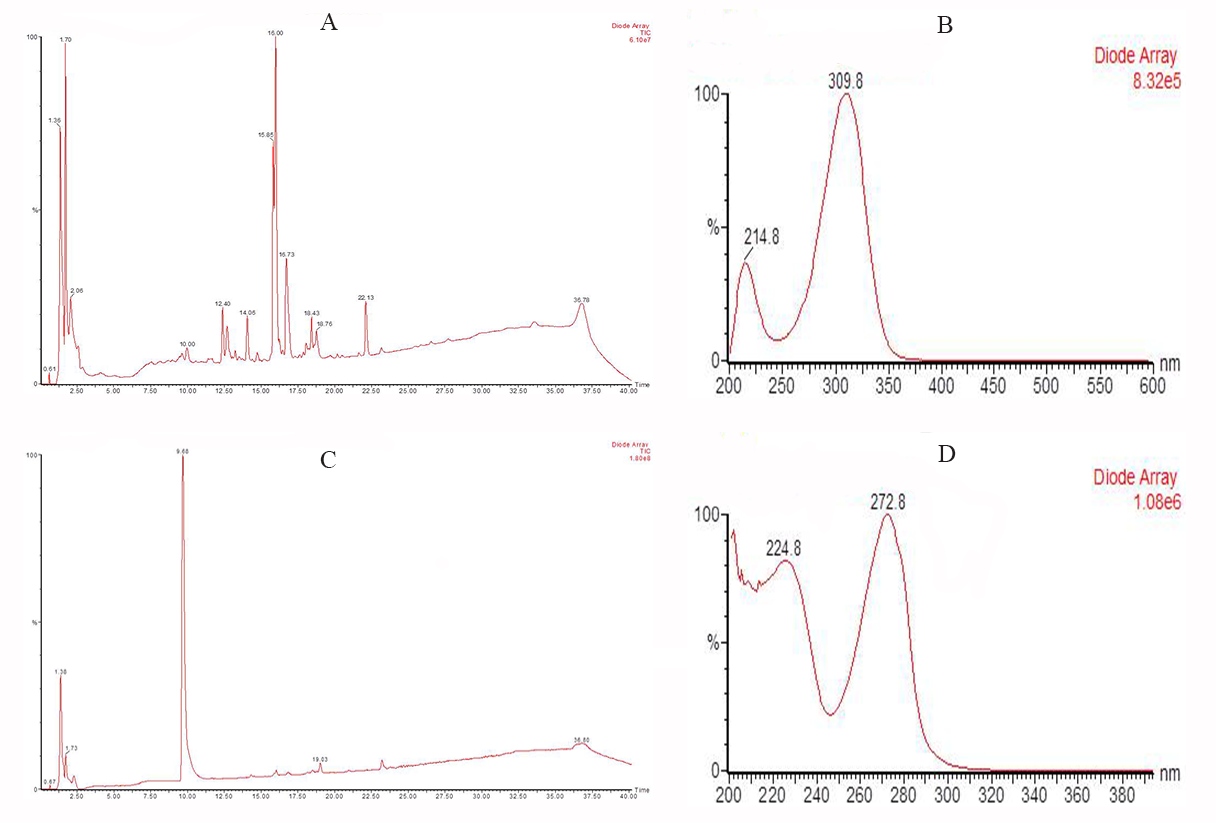


FIG S2 TIC and UV absorption spectrogram of the culture ﬁltrates of *F. proliferatum* 10R-7 in PDB medium and CDM(Czapek-Dox medium). A: TIC of the culture ﬁltrates of *F. proliferatum* 10R-7 in PDB medium. B: UV absorption spectrogram of the culture ﬁltrates of *F. proliferatum* 10R-7 in PDB medium. C: TIC of the culture ﬁltrates of *F. proliferatum* 10R-7 in CDM. D: UV absorption spectrogram of the culture ﬁltrates of *F. proliferatum* 10R-7 in CDM.


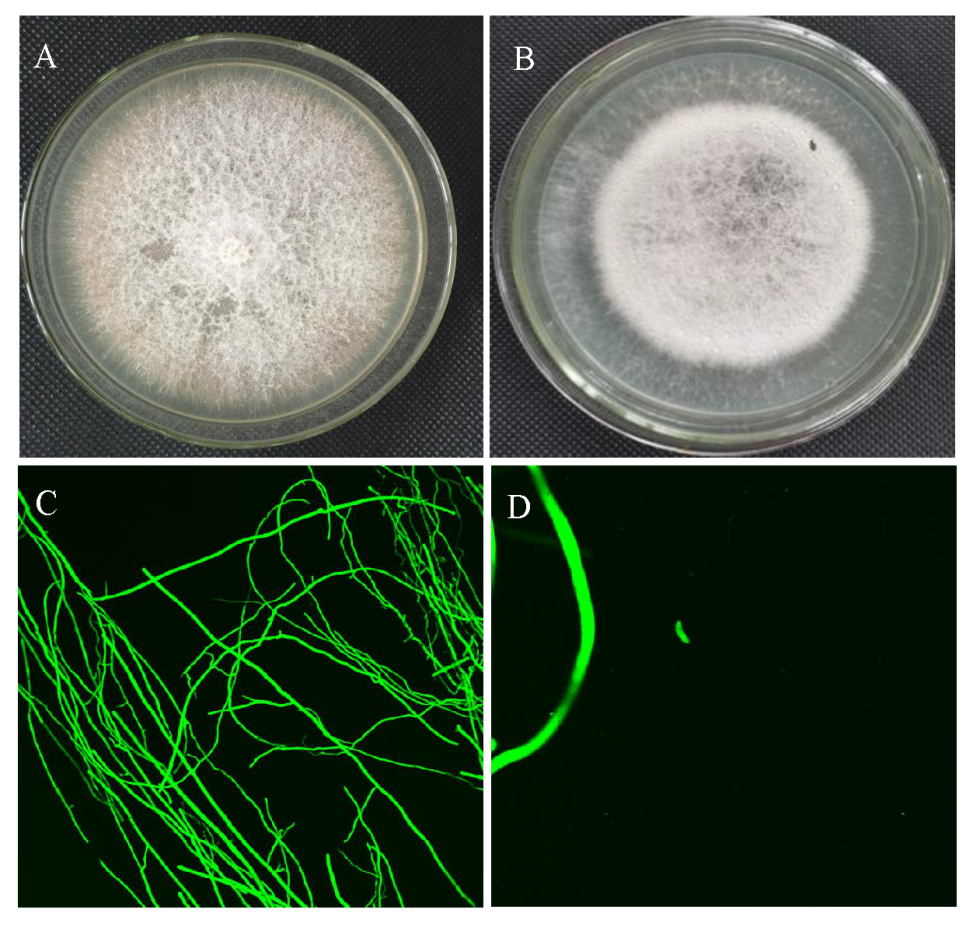


**Fig S3** The morphological characteristics of *F. proliferatum* 10R-7 and *F. proliferatum* 10R-7GFP. (A and B) The colony morphology of *F. proliferatum* 10R-7 and *F. proliferatum* 10R-7GFP, respectively. (C and D)Mycelial and conidial morphology of the proliferatum 10R-7GFP.
